# Supplementary material for: Estimated Covid-19 burden in Spain: ARCH underreported non-stationary time series
Source: BMC Med Res Methodol. 2023 Mar 28;23:75. doi: 10.1186/s12874-023-01894-9 (PMC10043853; doi:10.1186/s12874-023-01894-9)
Supplement: Supplementary file 1 — Additional file 1: Table S1. Root Mean Squared Error (RMSE) and Mean Absolute Percentage Error (MAPE) for the predicted number of cases in each Spanish CCAA. [file 12874_2023_1894_MOESM1_ESM.docx]

**SUPPLEMENTARY MATERIAL**

**Table S1. Root Mean Squared Error (RMSE) and Mean Absolute Percentage Error (MAPE) for the predicted number of cases in each Spanish CCAA.**

| **CCAA** | **RMSE** | **MAPE (%)** |
| --- | --- | --- |
| Andalucía | 9,161.06 | 9.11 |
| Aragón | 5,242.59 | 8.45 |
| Canarias | 3,898.54 | 7.88 |
| Cantabria | 1,671.47 | 9.72 |
| Castilla - La Mancha | 4,828.16 | 10.78 |
| Castilla y León | 6,675.46 | 6.74 |
| Catalunya | 27,638.43 | 7.24 |
| Ceuta | 287.52 | 9.77 |
| Nafarroa | 2,952.43 | 7.23 |
| País Valencià | 15,635.42 | 6.66 |
| Extremadura | 1,938.98 | 13.16 |
| Galiza | 6,902.80 | 7.87 |
| Illes Balears | 2,811.88 | 9.12 |
| La Rioja | 1,045.76 | 8.21 |
| Madrid | 12,766.85 | 7.40 |
| Melilla | 203.94 | 8.07 |
| Euskadi | 8,862.64 | 3.98 |
| Asturies | 2,491.66 | 7.30 |
| Región de Murcia | 4,161.40 | 7.75 |
